# Reirradiation clinical practice in gastrointestinal abdominal malignancies: an international reirradiation collaborative group (ReCOG) systematic review

**DOI:** 10.1016/j.ctro.2025.101033

**Published:** 2025-08-13

**Authors:** Nauman H. Malik, John P. Plastaras, Stefanie Corradini, Laura A. Dawson, Maria A. Hawkins, Kilian E. Salerno, Charles S. Mayo, Emma M. Dunne, Dorota Gabryś, Clemens Grassberger, Theodore S. Lawrence, Manju Sharma, Alanah M. Bergman, Dawn Owen, Ali Zaila, Soumon Rudra, Michael Velec, Donna H. Murrell

**Affiliations:** a Radiation Medicine Program, Princess Margaret Cancer Centre, University Hospital Network, and Department of Radiation Oncology, University of Toronto, Toronto, ON, Canada; b Penn Presbyterian Medical Centre, and University of Pennsylvania, Philadelphia, PA, USA; c University Hospital, Department of Radiation Oncology, LMU Munich, Germany; d Medical Physics and Biomedical Engineering, University College, London, UK; e Radiation Oncology Branch, National Cancer Institute, Bethesda, MD, USA; f Department of Radiation Oncology, University of Michigan, MI, USA; g Department of Radiation Oncology. BC Cancer Vancouver, British Columbia, Canada; h Department of Radiotherapy, Maria Sklodowska-Curie National Research Institute of Oncology Gliwice Branch, Poland; i Department of Radiation Oncology, University of Washington/Fred Hutchinson Cancer Center, Seattle, WA, USA; j Department of Radiation Oncology, University of California San Francisco, USA; k Medical Physics, BC Cancer-Vancouver, British Columbia, Canada; l Department of Radiation Oncology, Mayo Clinic Rochester, MN, USA; m King Faisal Specialist Hospital and Research Center (KFSHRC), Riyadh, Saudi Arabia; n Department of Radiation Oncology, Emory University School of Medicine, Atlanta, GA, USA; o Department of Oncology, Schulich School of Medicine & Dentistry, Western University, ON, Canada

**Keywords:** Reirradiation, Recurrence, Hepatocellular carcinoma, Review, Gastrointestinal cancers, Stereotactic body radiotherapy, Toxicity, Palliative radiotherapy, Salvage radiotherapy, Liver metastases

## Abstract

**Purpose:**

Reirradiation in abdominal malignancies has grown more common with advanced radiotherapy techniques. However, clinical use and implementation varies, and there remains limited consensus on best practices for reirradiation. In this systematic review, a multidisciplinary team treating gastrointestinal and hepatobiliary malignancies within the Reirradiation Collaborative Group (ReCOG) convened to review published literature on reirradiation in the abdomen to offer insights into patient selection, radiotherapy planning, risk management, and assessing knowledge gaps for future development of guidelines.

**Methods and Materials:**

A systematic search of Cochrane Central, CINAHL Plus, EMBASE, and PubMed up to August 30, 2024, was conducted as per Preferred Reporting Items for Systematic reviews and Meta-Analyses (PRISMA) framework. Data on patient characteristics, radiation doses, dose constraints, treatment outcomes, and toxicities were extracted. Where feasible, pooled weighted analyses were performed.

**Results:**

Thirty-three studies involving 1,264 patients met inclusion criteria: 30 were retrospective and 3 prospective. The median number of patients reported per study was 26 (range 2–245). Of the reported tumor sites, 718 patients had liver tumors and 277 pancreas, with smaller numbers of mixed/lymph node targets. Reirradiation doses, fractionation schemes, and dose constraints varied widely; only half of the studies provided explicit organ-at-risk constraints. Three studies included patients treated with palliative intent. Median overall survival ranged from 5.9 to 44 months, with a pooled weighted median OS of 19.6 months across 20 studies that reported it. One-year local control rates ranged from 19 % to 93 %, with severe (grade ≥ 3) toxicities typically reported in 5–15 % of patients, although one study reported 25 % lethal RILD in liver reirradiation.

**Conclusion:**

Reirradiation in abdominal malignancies appears to be able to achieve meaningful local control and survival in select patients, though heterogeneity in planning, dosing, and toxicity reporting remains a major challenge for establishing best practices. Standardized reporting of doses, constraints, and dose-volume relationships are needed to guide safe and effective reirradiation in this setting.

## Highlights

• This is review is a contemporary summary of published clinical experience in reirradiation of abdominal malignancies.
• There are heterogeneous clinical and technical practices, and limited reporting of data.
• We advocate for high quality, prospective data collection and standardized reporting to support clinical recommendations.

## Introduction

Abdominal malignancies, such as gastrointestinal (GI) and hepatobiliary cancers and metastases to the abdominal organs, pose a substantial therapeutic challenge in the reirradiation setting. Although radiotherapy is a commonly used treatment in the management of many patients with abdominal malignancies such as hepatocellular carcinomas, liver metastases, pancreatic cancer, cholangiocarcinoma, and targeting of abdominal lymph nodes, locoregional recurrences following initial treatment often leave clinicians and patients facing difficult decisions regarding further radiation treatment [1,2]. Prior radiotherapy course(s) may have exposed surrounding organs at risk (OARs) to significant doses and further radiation raises concern for increased risk of toxicity, particularly when targets approximate luminal GI organs [3]. Moreover, surgical options are often limited in this population, surgery can be challenging after prior radiotherapy, and surgery alone may not be sufficient without adjuvant RT to control microscopic spread of cancer. Systemic therapies have improved outcomes in many settings but are generally not curative on their own. Recurrences may be associated with a high likelihood of tumor-related locoregional morbidity. In these scenarios, therapeutic reirradiation is increasingly being explored despite the challenges of anatomic changes over time, inter-/intra-fraction motion of organs and tissues (e.g. from breathing, cardiac motion, variable bowel filling, peristalsis) that limit the accuracy of estimated cumulative doses to organ at risks (OARs) [[4], [5], [6], [7]]. The planning and delivery of reirradiation in these cases requires careful patient and tumor selection, motion management, and proactive management and mitigation of potential toxicity [8].

Recent technological advances, such as MR- and CT-guided adaptive radiotherapy, and widespread adoption of SBRT with motion management, have enabled more precise targeting and reduced dose to the surrounding tissues [[9], [10], [11], [12], [13], [14], [15]]. This has made reirradiation for abdominal malignancies feasible and fueled growing interest [16]. Despite these advances, there are no present standardized guidelines or consensus on best practices for reirradiation in abdominal malignancies [17,18]. This has led to considerable variability in clinical practice, thus highlighting the need for a comprehensive review of literature to inform evidence-based decision making and to identify the remaining gaps to help prioritize future initiatives to inform evidence-based guidelines.

This review, conducted under the auspices of the Reirradiation Collaborative Group (ReCOG), brings together radiation oncologists, radiation therapists, and medical physicist experts in treatment of gastrointestinal and hepatobiliary malignancies to examine published literature on abdominal reirradiation. We aim to compile data on reirradiation techniques, cumulative dose constraints, treatment outcomes and toxicities from a heterogeneous range of published literature, and to offer insights into optimizing patient selection, radiotherapy planning, and strategies to mitigate risks. The goal is to contribute to the development of guidelines in this evolving and challenging area.

## Materials and methods

A comprehensive search was performed of the following databases from inception until August 30, 2024: Cochrane Central, CINAHL Plus, EMBASE and PubMed. The systemic review was registered on PROSPERO (ID # CRD42025634890). Published data on clinical outcomes of reirradiation in the pancreas, liver, abdominal lymph nodes, gastrointestinal organs and other abdominal sites were identified. Details of the PRISMA search strategy are included in Appendix A, and the PRISMA checklists are outlined in Appendices B and C. Initial screening of titles and abstracts was done for relevance, followed by full text review to confirm eligibility. Controlled vocabulary (Medical Subject Headings [MeSH]) was systematically combined with free-text searches. Specifically, MeSH terms related to “reirradiation,” “liver neoplasms,” “pancreatic neoplasms,” “gastrointestinal neoplasms,” and “abdomen” were combined with relevant free-text keywords (e.g., “reirradiation,” “recurrent abdominal cancer,” “SBRT,” “salvage radiotherapy,” “hepatocellular carcinoma”). No limits were placed on publication year. One additional record was identified separately after initial search, reflected in the PRISMA flow diagram.

**Fig. 1:**
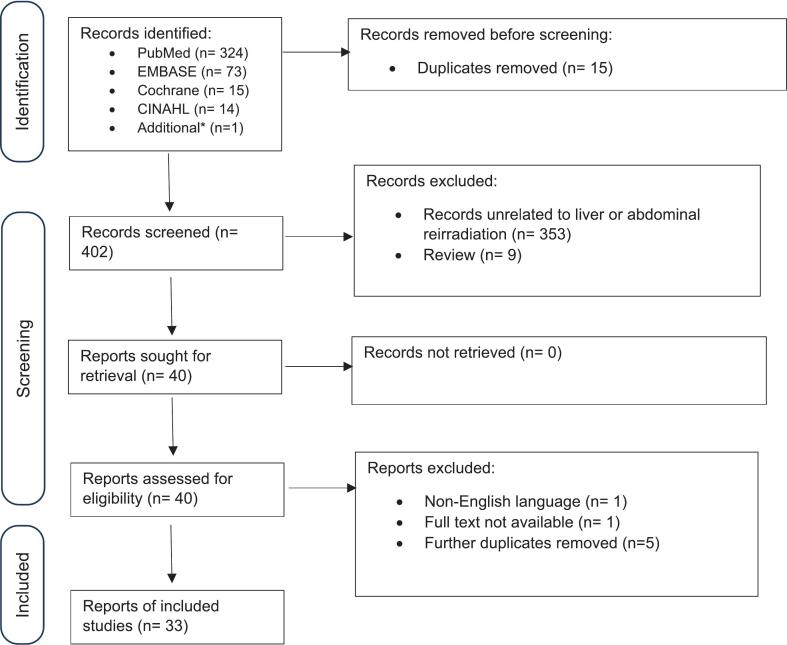
PRISMA flow diagram for the review. *Additional manuscript brought to authors’ attention separately.

The initial database search and abstract screening was conducted by one reviewer (NM). Studies meeting initial inclusion criteria underwent full-text review by all authors. Detailed data extraction and analysis were performed independently by two reviewers (DM and NM), with any discrepancies resolved through discussion and consensus among all authors. Both retrospective and prospective original studies were included in the search. Publications in non-English languages, duplicates, case reports, reviews, abstracts, and studies only on non-abdominal regions were excluded. If a study reported patients that included abdominal and non-abdominal sites, only the information relevant to abdominal sites was abstracted, if possible. References of included studies were also queried for potential eligible studies. Data collected included study design, patient characteristics, radiotherapy parameters (prior dose, reirradiation dose/fractionation, retreatment intervals, OAR constraints), clinical outcomes, and toxicities. Pooled weighted estimates for survival or local control endpoints were calculated by ∑(n_i_× estimate_i_)/∑n_i_, where n_i_ is the sample size of study i, and estimate_i_ is the reported outcome (e.g., median OS) for that study. A PRISMA workflow of the review is depicted in Fig. 1.

## Results

### Study selection and characteristics

A total of 33 studies met inclusion criteria, encompassing 1,264 patients receiving reirradiation for abdominal malignancies. Studies were predominantly retrospective (30 out of 33 studies; totaling 1,225 patients), with only three prospective studies (total 39 patients). The mean study enrollment was 38 patients (median 26, range 2–245). Primary anatomical sites irradiated included liver (n = 718), pancreas (n = 277), mixed abdominal sites (n = 79), and other locations (n = 190) such as lymph node or soft-tissue recurrences. Of the 13 liver studies, 9 reported on hepatocellular carcinoma (n = 617), 1 on liver metastases (n = 25), and 3 had a mixed cohort of patients including primary and metastatic tumors (n = 76). Treatment modalities across studies included 13 investigating stereotactic body radiotherapy (SBRT), 7 intensity-modulated radiotherapy (IMRT)/volumetric modulated arc therapy (VMAT), 4 proton beam therapy, 3 carbon ion therapy, 3 studies with mixed modalities, 2 utilizing MR Linac based radiotherapy, and 1 low dose rate (LDR) brachytherapy. Among these, 22 studies reported a primarily radical intent, 3 were palliative or mixed intent, and 8 did not specify intent. Study details are provided in Table 1.

**Table 1:** Included study design, and radiotherapy details.

Study (Year)	Design	Radiation modality	Patient Cohort Size	Indication(s)	Target(s)	Prior RT dose(s)	Reirradiation Prescription Dose(s)	Reirradiation BED/EQD2Gy	Reirradiation fractionation	Retreatment Interval in months (median, range)
Abusaris (2012)[43]	Retrospective study	IMRT/VMAT	27	NR	Pelvis, abdomen		34 (8–60) Gy	47 (24–150) EQD2Gy(10)	1–10	15 (2–52)
Boimel (2017)[31]	Prospective trial	Proton	15	definitive	Pancreas	50.4 Gy (30–59.4)	59.4 Gy (37.5–59.4 Gy)	NR		26.7 mo (7–90)
Chiloiro (2024)[46]	Retrospective study	MR Linac	25	definitive	Liver, metastases	43 Gy (24–50)	41 Gy (16–50)	mean 92 BED(10)	5	8.5 mo (2–28)
Chuong (2022)[40]	Retrospective study	MR Linac	8 out of 11	definitive	Pancreas	50 Gy (30–58.8)	40 (25–54) Gy	NR	5–36	NR
Dagoglu (2016)[25]	Retrospective study	SBRT	30	NR	Pancreas	50.4 (46.8–54) Gy	25 (24–36) Gy	NR	5 (3–5)	18 (4–316) mo
Gharzai (2023)[20]	Retrospective study	SBRT	47	Definitive	Liver, hepatocellular carcinoma	66 (17.2–150) EQD2Gy	NR	66 (19.8–––110) EQD2Gy	3 (2–5)	NR
Gkika (2019)[45]	Retrospective study	SBRT	24	NR	Liver, primary and metastatic tumors	46.5 (33–66) Gy	48 (27–66) Gy	NR	NR	14 mo
Goldsmith (2018)[29]	Retrospective study	SBRT	8 out of 42	definitive	Pancreas	NR	Mean: 26.77 Gy (19.33–113.39)/1 + fx	Mean: 50.3 (47.7–53.0) BED(10)	1+	NR
Hagiwara (2021)[41]	Prospective trial	Carbon Ion	21	definitive	Pancreas	Median: 52.8 (48–55.2) Gy (RBE)	Median: 52.8 (50.4–55.2) Gy (RBE)	NR	12	17 (6–95) mo
Hashimoto (2006)[47]	Retrospective study	Proton	27	definitive	Liver, hepatocellular carcinoma	72 Gy	66 Gy	NR	16	24.5 mo (3.3–––79.8)
Haque et al. (2009)[35]	Retrospective study	IMRT/VMAT	13	Mixed palliative and definitive	Pancreas, colon, cholangiocarcinoma, others	30–50.4 Gy	24–48 Gy	NR	180 cGy BID (hyperfractionateaccelerated)	26 (5–83)
Huang (2016)[42]	Retrospective study	SBRT	36	NR	Liver, hepatocellular carcinoma	30–60 Gy	10–60 Gy	NR	1.8–3 Gy per fraction	11 (1–47) mo
Hunt (2018)[36]	Retrospective study	IMRT/VMAT	24	Mixed palliative and definitive	pancreas, stomach, duodenum, liver, other	30–50.4 Gy	39 (15–45) Gy	NR	10–30	27.9 mo
Ito (2020)[44]	Retrospective study	IMRT/VMAT	113	definitive	Lymph nodes	NR	NR	59.7 (40.3–––101.4) EQD2Gy(10)	NR	8.5 mo
Kibe (2020)[55]	Retrospective study	SBRT	245	definitive	Liver, hepatocellular carcinoma	NR	35–40 Gy	NR	5	NR
Kimura (2020)[22]	Retrospective study	SBRT	81	definitive	Liver, hepatocellular carcinoma	NR	35–40 Gy	NR	4–5	18 mo (3–74)
Koong (2017)[26]	Retrospective study	SBRT	23	NR	Pancreas	50.4 (30–60) Gy	25 (20–33) Gy	NR	1–5	13 (2–32) mo
Lee (2016)[49]	Retrospective study	IMRT/VMAT	12	definitive	Liver, hepatocellular carcinoma	50 Gy (36–60)	50 Gy (36–––58.42)	NR	2.5 Gy − 9 Gy per fraction	20.3 (5.3–69.4) mo
Lee (2019)[34]	Retrospective study	IMRT/VMAT	42	Radical as doses of > 40 Gy/20F were used	hepatobiliary, abdominal node, pancreas	50 (30–60) Gy	45 (15–75) Gy	46.5 (13.8–125) EQD2Gy(10)	20 daily fractions	15.2 (3.8–69.3)
Lominska (2012)[27]	Retrospective study	SBRT	28	definitive	Pancreas	50.4 (41.4–70.2)	20–30 Gy	NR	3–5	NR
McDuff (2018)[19]	Retrospective study	Proton/IMRT/VMAT	49	NR	Liver, primary and metastatic tumors	NR	60 (59.1–83.3) EQD2Gy	72 (70.9–––100) Gy10	5–15	9 mo
Oshiro (2017)[48]	Retrospective study	Proton	83	definitive	Liver, hepatocellular carcinoma	NR	70.5 GyE (60–74)	NR	10–37	NR
Reddy (2022)[33]	Retrospective study	SBRT	27	definitive	Pancreas	33 Gy in 5 fractions or 50.4 Gy in 28 fractions	25–30 Gy	NR	5	19 mo (8.6–41.2)
Rwigema (2011)[50]	Retrospective study	SBRT	11 out of 71	definitive	Pancreas	NR	24 (18–25) Gy	NR	1	13.5 mo (0.8–47)
Ryan (2018)[28]	Retrospective study	SBRT	26 out of 51	definitive	Pancreas	NR	25 (25–33) Gy	NR	5	NR
Seol (2015)[23]	Retrospective study	IMRT/VMAT	45	Mixed palliative and definitive	Liver, hepatocellular carcinoma	NR	45 (35–55) Gy	44 (31.25–93.75) EQD2Gy(10)	3 (2–15) daily fractions	13.8 (1–58.1) mo
Shen (2021)[30]	Retrospective study	SBRT	24	definitive	Pancreas	35.5 Gy in 5–7 fractions	32 Gy	51.4 Gy BED (37.5–72 Gy)	5–8	13 (6–29) mo
Shirai (2019)[39]	prospective trial	Carbon Ion	3 out of 22	definitive	Liver, primary and metastatic tumors	NR	60 Gy	NR	4	14.5–––28.2 mo
Sutera (2018)[32]	Retrospective study	SBRT	38	definitive	Pancreas	50.4 Gy (30–59.4)	24.5 Gy (24–30)	NR	1–3	NR
Tomizawa (2023)[21]	Retrospective study	Carbon Ion	41	definitive	Liver, hepatocellular carcinoma	NR	52.8–60 Gy RBE	NR	4–12	NR
Wild (2013)[24]	Retrospective study	SBRT	18	definitive	Pancreas	50.4 (45–50.4) Gy	25 (20–27) Gy	NR	5 fractions consecutive	NR
Yao (2015)[37]	Retrospective study	LDR brachytherapy	17	definitive	Lymph nodes	NR	D90 100–198 Gy (median 126.5 Gy)	NR	NR	NR
Yoshida (2023)[38]	Retrospective study	IMRT	33	definitive	Lymph nodes	58.4 (40.0 – 83.3) EQD2Gy(10)	NR (some received 50 and 60 Gy)	70.4 (49.6 – 93.3) EQD2Gy(10)	NR (at least some patients reported to receive 5 and 20 fraction regimens)	15 (5 – 82) months

### Initial radiation and reirradiation parameters

Reirradiation doses varied substantially across the studies, and reporting on prior radiotherapy doses, reirradiation dose fractionation, retreatment interval, and prescription isodose line was heterogeneous.

Twenty studies explicitly detailed prior delivered doses, all from prior external beam radiotherapy. No study reported initial use of brachytherapy or Yttrium-90 therapy. Previously delivered doses were commonly in the 30–60 Gy range for conventionally fractionated regimens, and a subset had received prior SBRT. Reirradiation doses were similarly heterogeneous: including moderate total doses (20–40 Gy in 5–10 fractions) and SBRT regimens (e.g., 25–60 Gy in 1–5 fractions). Eighteen studies provided some cumulative and/or reirradiation planned OAR constraints, including seven studies that reported planned cumulative OAR dose constraints. No studies provided estimates of cumulative dose and tumor control probability or cancer outcome modeling to targets.

Among the 33 studies reviewed, retreatment intervals were inconsistently reported, with 21 studies documenting a median or mean time between primary radiation and reirradiation, ranging from 1 month to 316 months. Several retrospective cohorts did not specify the maximum cumulative doses received by tissues or volumes of targets.

Where reported, prescriptions expressed in equieffective dose (α/β = 10) for the reirradiation course alone were commonly 45–60 EQD2Gy, but ranged from 13 −150 EQD2Gy, reflecting substantial heterogeneity in patient selection and treatment intent. Most reirradiation SBRT protocols for liver or pancreas delivered doses in the 30–60 Gy range over 3–5 fractions, whereas studies with more conventional regimens generally employed 40–60 Gy in 20–30 fractions.

Only 4 studies reported prescription methods, such as prescription to an isodose line, ranging from 60 % to 100 %. Treatment setup and image guidance details were seldom reported. Systemic therapy and other treatment details were also rarely reported, and most studies did not report on recovery factors- either assumed (e.g. during planning) or calculated (e.g. based on observed outcomes). None of the studies reported planned versus delivered doses or strategies and methods used to attempt to separate prior irradiated volumes from reirradiation volumes. Details of dose constraints for organs at risk across studies are provided in Table 2. Bowel was the organ at risk most often included for evaluation. Some studies reported constraints for reirradiation and only a few reported cumulative constraints. There does not appear to be consensus on dose constraint metrics used nor the limit values.

**Table 2:** Organ at Risk (OAR) dose constraints in included studies. Constraints are for the reirradiation plan only, unless otherwise indicated; only 7 studies reported dose constraints for accumulated dose. Studies not included in this table did not report constraints.

Study (Year)	Liver	Kidneys	Stomach/Duodenum/Bowel	Spinal cord	Additional OARs/notes
Abusaris (2012)[43]	—	—	*CUMULATIVE DOSE: Rectum/bowel Dmax < 110 EQD2Gy(3)	—	*CUMULATIVE DOSE: Bladder Dmax < 120 EQD2Gy(3)
Gharzai (2023)[20]	Mean < 9 Gy (3 fx)/<13 Gy (5 fx), CV15Gy > 700 cc (3fx)/CV19Gy > 700 cc (5fx)	—	—	—	“Cold volume” approach for healthy liver sparing
Goldsmith (2018)[29]	V15Gy ≤ 50 % (3fx) V21Gy ≤ 30 % (3fx)	V15Gy ≤ 35 % (3fx)	Small bowel D0.035 cc ≤ 25.2 Gy (3fx) Stomach/duodenum D0.035 cc ≤ 22.2 Gy (3fx)	D0.035 cc ≤ 21 Gy (3fx) D0.1 cc ≤ 18 Gy (3fx)	—
Hagiwara (2021)[41]	—	—	*CUMULATIVE DOSE: D2cc GI tract ≤ 60 Gy (RBE)	*CUMULATIVE DOSE Dmax ≤ 40 Gy (RBE)	—
Huang (2016)[42]	Mean ≤ 23 Gy_2_	Median < 20 Gy_2_	Duodenum/Stomach: V50 Gy_2_ < 1 cc	*CUMULATIVE DOSE: <60 Gy_2_	—
Ito (2020)[44]	—	For high precision RT:V50EQD2Gy (3) < 33 %	For 3DCRT: stomach & small bowel Dmax < 52 EQD2Gy(3) large bowel Max < 62 EQD2Gy(3) For high precision RT: Stomach & small bowelV54EQD2Gy (3) < 3cc Large bowel V64EQD2Gy(3) < 3cc	For 3DCRT: Dmax < 50 EQD2Gy(3) For high precision RT: Dmax < 52 EQD2Gy(3)	—
Kimura (2020)[22]	—	—	Stomach/Bowel D10cc < 25 Gy (4-5fx)	Dmax < 25 Gy (4-5fx)	—
Koong (2017)[26]	—	—	Dmax < 30 Gy; V25Gy ≤ 1 cc; V20Gy ≤ 3 cc; V15Gy ≤ 5 cc (stomach/bowel/duodenum for 5fx)	—	—
Lee (2016)[49]	*CUMULATIVE DOSE: Mean remaining liver dose ≤ 30 Gy, ≥700 cc spared	—	*CUMULATIVE DOSE: D2cc ≤ 50 Gy (small/large bowel) D2cc ≤ 45 Gy (duodenum/stomach)	—	—
McDuff (2018)[19]	V30 < 30 % Dmean < 8 Gy (5-6fx) or < 24 Gy (15 fx)	Combined: V15Gy < 66 %; if single kidney: V7Gy < 90 % (5–6 fx) Combined: V18Gy < 30 %; V10Gy < 60 % (15 fx)	Stomach + 5 mm, Esophagus + 5 mm, Bowel + 5 mm: V30Gy < 0.5 cc (5-6fx) Stomach + 5 mm, Esophagus + 5 mm: Dmax 42 Gy (15 fx) Bowel + 5 mm: Dmax 45 Gy (15 fx)	Cord + 5 mm: Dmax 22 Gy (5-6fx) or 30 Gy (15fx)	Heart + 5 mm: Dmax 20 Gy (5-6fx) or V40Gy < 10 % (15 fx) Chest wall: V40Gy < 2 cc (5-6fx) or V60Gy < 2cc (15 fx) *CONSTRAINTS APPLY TO CUMULATIVE DOSE IF TREATMENT DURATION ≤ 12 months
Oshiro (2017)[48]	—	—	Alimentary tract ≤ 50 GyE	≤50 GyE	—
Reddy (2022)[33]	*CUMULATIVE DOSE: >700 cc < 30 Gy BED(3)	*CUMULATIVE DOSE: 50 % each < 21.6 Gy BED(3)	Stomach/small bowel: V25Gy < 5 cc, V15Gy < 20 cc, Dmax < 30 Gy	*CUMULATIVE DOSE: Dmax < 75 Gy BED(3) accounting for 50 % recovery	
Rwigema (2011)[50]	Dmax 8.4 Gy	R kidney 2.9 Gy, L kidney 3.5 Gy	Bowel/stomach Dmax 15.1 Gy	Observed Dmax 1.8 Gy	Highest observed doses reported, no formal constraint
Seol (2015)[23]	V50% < 50 % (liver volume)	—	—	—	—
Shen (2021)[30]	*CUMULATIVE DOSE: Mean 5.29 Gy	*CUMULATIVE DOSE: D < 2/3 ∼ 3 Gy (both kidneys)	*CUMULATIVE DOSE: Duodenum Dmax ∼ 35.5 Gy, D5cc ∼ 19.5 Gy Bowel Dmax ∼ 44.4 Gy, D5cc ∼ 29.9 Gy Stomach Dmax ∼ 44.2 Gy, D10cc ∼ 26.7 Gy	*CUMULATIVE DOSE: Dmax 8.54 Gy, D0.35 cc 7.93 Gy	Median cumulative doses only
Tomizawa (2023)[21]	V20(RBE) < 35 %	—	Dmax(stomach/bowel/duodenum) = 42 Gy(RBE)	—	—
Wild (2013)[24]	D50% <12 Gy	—	Duodenum: V15Gy < 9 cc, V20Gy < 3 cc, V33Gy < 1 cc Stomach: D50% <12 Gy, V33 < 1 cc	V8Gy < 1 cc	—
Yao (2015)[37]	—	—	bowel dose Dmax 60 Gy	Max 36 Gy	Median doses
Yoshida (2023)[38]	—	—	Duodenum, intestines Mucosal layer D1cc < 70 EQD2Gy(3) Circumference D5cc < 50 EQD2Gy(3) *CUMULATIVE DOSE: Mucosal layer D1cc < 120 EQD2Gy(3) Circumference D5cc < 100 EQD2Gy(3)	—	—

**Dxx** = dose to xx% or xx cc of the volume; **Vxx** = volume receiving at least xx Gy; **Gy2** = constraints in 2 Gy/fraction equivalent; **RBE** = relative biological effectiveness correction for carbon ion/proton; **BED**/**EQD2Gy** = biologically effective dose/equivalent dose in 2 Gy fractions with alpha/beta ratio in parentheses.

### Outcomes

Reporting of outcome endpoints was highly variable, and some studies did not include any description of endpoints. Median OS was reported in 19 studies, and ranged from 5.9 to 44 months, with a pooled weighted average across those studies of 19.6 months. Across the 5 liver studies reporting median OS (n = 263) [[19], [20], [21], [22], [23]], median OS was 25.3 months; across the 10 pancreas studies (n = 237) [[24], [25], [26], [27], [28], [29], [30], [31], [32], [33]], median OS was 16.1 months; across 3 studies reporting mixed targets (n = 79) [[34], [35], [36]], median OS was 13.2 months, and 1 study targeting lymph nodes (n = 17) [37] reported a median OS of 10 months, with Yoshida (2023) [38] only reporting 2-year OS rate of 45.5 %, and was therefore excluded from pooled analyses. Four liver studies with cohorts comprising only recurrent hepatocellular carcinoma reported a pooled median OS of 27.9 months. Fifteen studies reported 1-year OS, ranging from 17.5 % to 85 %.

Most studies reported different local efficacy endpoints (local control [LC] vs freedom from local progression [FFLP] vs local failure [LF]), with variable estimates ranging from 6 to 36 months. Overall, 1-year LC rates ranged from 18.8 % to 93.4 % across all included studies. Only 3 liver studies reported 1-year LC (n = 93) with a range from 39 % to >90 % [19,21,39], with a pooled estimate of 64 %; it was not feasible to separate 1-year LC for primary and metastatic liver tumors. Ten pancreas studies reported 1-year LC or FFLP (n = 214) ranging from 37 % to 89 % [[24], [25], [26],^28^,^29^,[31], [32], [33],^40^,^41^], with a pooled estimate of 66 %.

### Toxicities

Toxicity profiles were reported across all studies, with gastrointestinal (GI) toxicities being the most commonly reported toxicities. Of the liver studies that reported Common Terminology Criteria for Adverse Events (CTCAE) acute toxicities (n = 219) [19,20,23,34,42], 122 patients (56 %) were reported to experience grade 1–2 events. Grade ≥3 GI toxicities ranged from 0 to 7 % in most cohorts, except in the study by Kimura (2020) [22] which reported all toxicities in aggregate. Of pancreas studies, grade 1–2 GI toxicities were not always reported, with clearest reporting in Boimel (2017) [31], Shen (2021) [30], Chuong (2022) [40], and Reddy (2022) [33] reporting grade 1–2 toxicity in 87 %, 21 %, 9 %, and 41 % respectively. For mixed studies, reported rates of grade 1–2 acute toxicities were 5–81 %, and for grade ≥3 toxicities were 0–8 % [[34], [35], [36], [37],^43^,^44^]. Grade ≥3 toxicities included perforated viscus, bowel obstruction, GI bleeding, duodenal stricture, duodenal ulcer, bile duct stenosis, diarrhea, cholangitis, rib fracture, and radiation induced liver disease (RILD).

Among the 12 liver-dominant studies that reported late toxicities (n = 512) [[19], [20], [21], [22], [23],^34^,^42^,[45], [46], [47], [48], [49]], the rates of grade ≥3 toxicity reported were 0–30 %, with a total of 45 events reported, for a pooled rate of 8.8 %. From this, Huang et al. (2016) [42] was an outlier and strongly influenced the rate with nine grade 5 radiation-induced liver disease (RILD) events out of 36 patients (25 %)?. Among the thirteen pancreas studies (n = 215) [[24], [25], [26], [27], [28], [29], [30], [31], [32], [33],^40^,^41^,^50^], rates of grade ≥ 3 toxicity ranged from 0-17 %, with a total of 22 events, for a pooled rate of 10.2 %. Studies that combined multiple sites or focused on nodal recurrences [[34], [35], [36], [37],^43^,^44^] reported a range of late grade ≥3 toxicities of 0–16.7 %.

Regarding radiotherapy modalities, SBRT for liver tumors was associated with variable late grade ≥3 toxicity rates of 0–30 %, while pancreas rates were 5–15 %. In studies treating with conventional dose and fractionation, late grade ≥3 events ranged from 0-16 %. For particle therapy studies, proton series reported rates of 0–18 % for late grade ≥3 toxicity in liver tumors, and up to 13 % in pancreatic series, while carbon ion series reported rates of 0–10 %. Details on usage of image guided radiotherapy, motion management and planning strategies to mitigate toxicity outcomes was not reported. Details of reported outcomes and toxicities are outlined in Table 3.

**Table 3:** Key reported outcomes and toxicities across included studies.

Study (Year)	Key Reported outcomes	Key reported toxicities
Abusaris (2012)[43]	1-yr LC 64 %, 2-yr LC 53 %	G2 pain 7 %, G2 nausea 11 %, G1 skin 7 %, others ∼ 4 %
Boimel (2017)[31]	Median OS 16.4 mo, 1-yr in-field FFS 87 %, 1-yr OS 67 %	87 % G1–2 acute, 13 % G4–5 (1 duodenal ulcer, 1 bowel perforation & death)
Chiloiro (2024)[46]	1-yr OS 85 %, 1-yr PFS 55 %	No reported toxicities, no RILD
Chuong (2022)[40]	1-yr OS 17.5 %, 1-yr FFLP 88.9 %	9.1 % G2 acute, 0 % G ≥ 3
Dagoglu (2016)[25]	Median OS 14 mo, 1-yr LC 78 %	G3 acute bleed/pain 11 %, late G3 bowel obstruction 7 %
Gharzai (2023)[20]	Median OS 17 mo	G1–2: 25 %, no G ≥ 3
Gkika (2019)[45]	NR (no outcomes reported)	No G2 + toxicities observed
Goldsmith (2018)[29]	Median OS 8.4 mo, 1-yr FFLP 43 %, PFS 5.9 mo	8 % acute G3, 11 % late G4 (duodenal stricture, GI bleed)
Hagiwara (2021)[41]	1-yr LC 53.5 %, 1-yr PFS 24.5 %, 1-yr OS 48.7 %	5 % G3 acute duodenal stenosis, 0 % late ≥ G3
Haque et al. (2009)[35]	Median OS 14 mo, 1-yr OS 62 %, 1-yr freedom from LF 50 %	8 % G2 acute, 8 % G3 (progressed to late G4 requiring surgical revision)
Hashimoto (2006)[47]	5-yr LC 86.3 %; 23/27 died by median FU 62.2 mo (no explicit median OS)	18 % G ≥ 3 (acute hepatic failure, rib fracture, cholangitis)
Huang (2016)[42]	NR (OS not reported); RILD 36 %	9 patients died of RILD (25 % lethal)
Hunt (2018)[36]	Median OS 14 mo, 1-yr OS 50 %, 1-yr LPFS 38 %	G1–2 GI 54 %, 8 % G3 acute
Ito (2020)[44]	2-yr OS 63.1 %, 2-yr LC 59.7 %, 2-yr PFS 19.4 %	G3 diarrhea 3 %, stomachache 1 %, anorexia 1 %
Kibe (2020)[55]	3-yr LC 1.4–5 %, 3-yr OS 66.1 %	No RILD
Kimura (2020)[22]	3-yr LR 4.5 %, median OS 44 mo	17.3 % G3
Koong (2017)[26]	Median OS 27.5 mo, 1-yr freedom from LF 81 %	Late G2–3: 26.1 %
Lee (2016)[49]	4 deceased, 8 alive (no explicit OS)	1 perforated duodenal ulcer (≥G3)
Lee (2019)[34]	Median OS 12.5 mo; In-field FFS 9.2 mo	G1–2: 81 %, G3 acute: 7 %, late G3–4: 9 %
Lominska (2012)[27]	Median OS 5.9 mo, 1-yr OS 18 %	1 G2 bowel obstruction, 1 G3 gastric perforation
McDuff (2018)[19]	Median OS 14 mo; 1-yr LF 61 %	G1–2: 85.7 %, G3: 4.1 %, 0 % G4–5
Oshiro (2017)[48]	ReRT outcomes not reported	0 % G ≥ 3
Reddy (2022)[33]	Median OS 18.3 mo, 1-yr OS 73.1 %, 2-yr OS 33.6 %, 1-yr LPFS 62.9 %, 2-yr LPFS 27.2 %	G1–2 fatigue 41 %, G3 abdo pain 4 %, G3 SBO 4 %
Rwigema (2011)[50]	1-yr OS 58.4 %, 1-yr FFLP 18.8 % (*not specific to reRT subset*)	4 % acute G3 in entire SBRT group; 0 % late
Ryan (2018)[28]	Median OS 11 mo, 1-yr FFLP 37 %, PFS 7 mo	4 % acute G3 bowel obstruction, 12 % late G3–4 (obstruction, GI bleed)
Seol (2015)[23]	1-yr OS 57 %, 2-yr OS 38 %, median OS 11.2 mo	G1–2 GI: 41.9 %
Shen (2021)[30]	Median OS 14 mo (localized)/6.5 mo (mets)	4 % G3 acute (vomiting, diarrhea), no G4–5
Shirai (2019)[39]	1-yr LC 71 %, 2-yr LC 60 %, 1-yr OS 76 %, 2-yr OS 67 %	0 % ≥G2
Sutera (2018)[32]	Median OS 26.6 mo, 1-yr OS 87 %, 2-yr OS 53 %, 2-yr LC 82 %	G2 + 18.4 %, G3 + 10.5 %, 1 G4 duodenal stenosis
Tomizawa (2023)[21]	Median OS 27 mo; 1-yr LC 93.4 %, 2-yr LC 83 %, CSS 59.5 %	∼7.3 % G3 non-hematologic (bile duct stenosis, GI bleeding)
Wild (2013)[24]	Median OS 8.8 mo, PFS 3.7 mo, 1-yr local control 62 %	28 % G2 GI (fatigue, pain, anorexia, nausea, diarrhea), no G ≥ 3
Yao (2015)[37]	Median OS 10 mo, no local failures	No toxicities
Yoshida (2023)[38]	2-yr OS 45.5 % overall, 63.6 % localized RT field, 9.1 % multiple RT fields	12.1 % G3 or higher GI toxicity, 2 G3 duodenal obstruction, 1 G3 rectal ulcer and radiation enteritis, 1 G5 duodenal ulcer and bleeding

**OS** = overall survival; **LC** = local control; **LF** = local failure; **FFLP** = freedom from local progression; **LR** = local recurrence; **PFS** = progression-free survival; **RILD** = radiation-induced liver disease; **Gx** = CTCAE grade x.

## Discussion

Reirradiation in gastrointestinal abdominal malignancies represents an evolving area of practice driven by a need for effective local therapeutic options for local control in patients who have already undergone a prior course of radiation therapy. Our review identified 32 studies, predominantly retrospective, illustrating considerable heterogeneity in patient selection, dose prescription, fractionation, and inconsistent reporting of outcomes and toxicities. Despite a lack of high-level evidence on best practices with reirradiation, our contemporary review shows that publications in abdominal reirradiation appear to be growing over time. The marked heterogeneity in patients, timing of re-treatment, radiation doses and fractionation schedules, and outcome reporting precluded a formal *meta*-analysis [51].

A broad range of pooled outcomes were seen in our review, and individual patient level data was not available. It is challenging to draw conclusions on cumulative dose/volume/outcome relationships, due to lack of consistent reporting of endpoints and relevant clinical considerations. Furthermore, dose–response relationships were not clearly described in the studies that included some dosimetric information. Most studies did report on toxicity: acute grade 1–2 toxicities were common, while late severe (grade ≥ 3) toxicity rates were uncommon, ranging from 5 to 15 % of patients, with one outlier study, reporting a 25 % rate of fatal RILD in liver cancer patients re-irradiated [25]. The authors reported pre-treatment Child-Turcotte-Pugh score ≥6 predicted grade 5 RILD in multivariable analyses with odds ratio 72.6 (95 % confidence interval 3.7–1442.3, p < 0.01), emphasizing the strong importance of pretreatment liver function impacting risk of liver toxicity, generally overshadowing the impact of dosimetric details. Reirradiation interval, CTV or PTV volumes, mean cumulative liver dose, mean cumulative prescribed dose, and mean normal liver dose all were not associated with grade 5 RILD. The authors concluded that pretreatment residual liver function played a crucial role in determining tolerance to reirradiation and survival after treatment, consistent with studies of liver irradiation in the upfront setting [56]. Eighteen studies reported either cumulative or reirradiation course OAR constraints, which varied widely. Moreover, studies did not report association of fractionation schemes, image guidance, and motion management on toxicity.

The present review highlights that optimal treatment strategies are unclear from the literature, given the heterogeneity of reported information such as patient selection, treatment intent, details on prior treatments, treatment planning constraints and delivery details, details on radiotherapy doses, clinical confounding factors and outcomes and toxicities. We strongly advocate that researchers and clinicians publish their abdominal reirradiation data and outcomes in accordance with the ESTRO-EORTC consensus guideline, such that systematic review and radiobiological modelling efforts akin to QUANTEC, HyTEC, and PENTEC can be performed [52]. Additionally, our review builds on the important groundwork by a previous systematic review in 2019 by the Re-irradiation Working Group of the Association of Radiotherapy and Clinical Oncology (AIRO) [16]. The AIRO review provided valuable insights into abdominal reirradiation practices but was limited by fewer included studies (16 studies, 408 patients) and less comprehensive reporting of technical aspects and cumulative dosimetry. Our current systematic review builds substantially upon this prior work by integrating data from a larger patient cohort (32 studies, 1231 patients), incorporating recent technological advances in radiotherapy delivery and planning, and emphasizing the need for standardized reporting criteria based on ESTRO-EORTC recommendations. Together, these efforts represent significant progress toward developing comprehensive, evidence-based guidelines for safe and effective abdominal reirradiation.

Our present study has some limitations. Individual patient-level data were not available. Included studies report outcomes across multiple tumor histologies and different abdominal sites, with varying prior therapies, missing clinical, technical and dose volume details, and diverse fractionation schemes. There is also a lack of reported information on brachytherapy and Yttrium-90 radioembolization in abdominal malignancies, although a recent abstract of a phase I trial explored single photon emission computed tomography (SPECT) to allow preferential sparing of functional liver volume after prior external beam radiotherapy and Yttrium-90 radioembolization, and report with a reirradiation prescription of 45–67.5 Gy in 15 fractions, ≥500 cc of functional liver sparing from >22 Gy resulted in only 1 of 13 patients experiencing dose limiting toxicity [53]. Regarding brachytherapy, one study showed carefully selected patients can undergo multiple single-fraction high dose rate brachytherapy treatments to overlapping liver regions without excessive toxicity, provided the irradiated volume remains small [54]. A formal risk-of-bias assessment was not performed due to study heterogeneity, and future reviews would benefit from a structured bias evaluation given the variability in methodology, patient selection, reporting quality and treatment details across included studies.

In the present review, pooled values were reported from aggregate outcomes rather than individual patient data, so should therefore be interpreted as broad descriptors rather than definitive benchmarks [51]. Patients considered for reirradiation in the abdomen are heterogeneous group given the various types of malignancies and metastases that may present in the region, and they undergo a broad array of prior treatments, often in combination. A range of radiation prescriptions were used across studies in both radical and palliative intents, and radiotherapy dose and planning data were inconsistently reported across studies. In many studies, no or limited data on cumulative organs-at-risk constraints were reported, and details of motion management, image guidance, dose accumulation and fusion were rarely mentioned. Even taking the studies where constraints were provided, it is not possible from our review to define meaningful organ-specific dose/volume/outcome data. Additionally, data on systemic therapies, surgery, and interventional procedures was not reported by all studies or their timing relative to reirradiation. Nevertheless, our review may provide guidance for investigators interested in identifying collaborators to retrospectively extract and pool patient-specific data across multiple institutions to obtain better data in the reirradiation setting.

While high quality data are presently lacking, there are ongoing efforts to build reirradiation platforms globally and thoroughly collect data. The ReCare study is a prospective, observational cohort on high-dose reirradiation in the E2-RADIatE platform (NCT03818503). In the meantime, a patterns of practice international survey on reirradiation in clinical practice showed that presently the most common sites for reirradiation are brain, pelvis, and head and neck, while only 39 % of respondents performed reirradiation in the abdomen [17]. There are no published guidelines for abdominal reirradiation which may contribute to the lower uptake of abdominal repeat RT compared to other anatomic sites. In the ReCare study, the most common abdominal sites for reirradiation were lymph node recurrences, liver metastases, adrenal metastases, and locally recurrent pancreatic cancer respectively. The most common treatment goal was prolonging local control, and persistent grade 3 or higher radiation-induced toxicity was the leading contraindication to reirradiation [17].

Considering the findings from this review and existing data, it is evident that reirradiation practices in abdominal malignancies vary significantly across institutions and regions, and there is a paucity of high-level evidence guiding clinical decision making. This heterogeneity in treatment approaches, dose constraints, radiotherapy planning and delivery limit comparisons to improve patient outcomes. We recommend that future studies incorporate recommendations from the European Society for Radiotherapy and Oncology (ESTRO) and European Organization for Research and Treatment of Cancer (EORTC) consensus on reirradiation definition, reporting, and clinical decision making framework to facilitate cross-study comparisons and creation of high level guidelines [8]. The collection, recording, and reporting of dose/volume/outcome data for patients undergoing reirradiation is challenging. Indeed, the lack of completeness and clarity in many reports regarding these data elements is perhaps a “symptom” of these challenges. Pending reports from ReCOG and other groups will provide detailed guidance regarding some of these issues for future studies. For example, the following elements are generally considered: (1) essential patient and tumor characteristics (performance status, OAR baseline function when possible (e.g. liver), prior therapies, target volume), (2) all relevant prior RT courses (dose, fractionation, interval in physical units and/or EQD2Gy/BED, specifying α/β and any discount/tissue-recovery factors), (3) current reirradiation parameters (total dose, fractionation, modality, motion management, organ-at-risk constraints in physical or EQD2Gy/BED), (4) methods of summing or registering prior and current doses (rigid vs. deformable), and (5) separate acute versus late toxicities with information on grading, alongside consistent endpoints (local control, progression-free survival, overall survival). Future studies systematically addressing these highlighted gaps will be essential to inform evidence-based practice and optimize outcomes in abdominal reirradiation.

## Conclusion

Reirradiation in abdominal malignancies appear to be able to achieve meaningful local control and overall survival for appropriately selected patients, yet wide variability in dose, fractionation, and reporting hampers cross‐study comparisons and consensus. Moving forward, standardizing data collection, including detailed reporting of prior and current radiotherapy parameters, cumulative OAR exposures, and separate acute and late toxicities, will be key to developing rigorous, evidence‐based guidelines.

## CRediT authorship contribution statement

**Nauman H. Malik:** Conceptualization, Investigation, Formal analysis, Writing – original draft. **John P. Plastaras:** Conceptualization, Supervision, Writing – review & editing. **Stefanie Corradini:** Conceptualization, Writing – review & editing. **Laura A. Dawson:** Conceptualization, Supervision, Writing – review & editing. **Maria A. Hawkins:** Conceptualization, Writing – review & editing. **Kilian E. Salerno:** Writing – review & editing. **Charles S. Mayo:** Writing – review & editing. **Emma M. Dunne:** Writing – review & editing. **Dorota Gabryś:** Writing – review & editing. **Clemens Grassberger:** Writing – review & editing. **Theodore S. Lawrence:** Writing – review & editing. **Manju Sharma:** Writing – review & editing. **Alanah M. Bergman:** Writing – review & editing. **Dawn Owen:** Writing – review & editing. **Ali Zaila:** Writing – review & editing. **Soumon Rudra:** Writing – review & editing. **Michael Velec:** Writing – review & editing. **Donna H. Murrell:** Conceptualization, Supervision, Validation, Writing – review & editing.

## Declaration of competing interest

The authors declare the following financial interests/personal relationships which may be considered as potential competing interests: Dorota Gabrys reports a relationship with Varian Medical Systems Inc that includes: speaking and lecture fees. Soumon Rudra reports a relationship with InformAI that includes: consulting or advisory. Laura Dawson reports a relationship with AstraZeneca that includes: consulting or advisory. Laura Dawson reports a relationship with Elekta that includes: consulting or advisory. Laura Dawson reports a relationship with Merck that includes: funding grants. If there are other authors, they declare that they have no known competing financial interests or personal relationships that could have appeared to influence the work reported in this paper.

## Figures and Tables

**Table:** 

Search number	Query	Results
7	#5 OR #6	324
6	((“re irradiation”[MeSH Terms] OR “re irradiation”[All Fields] OR “reirradiation”[All Fields] OR “reirradiate”[All Fields] OR “reirradiated”[All Fields] OR “reirradiating”[All Fields] OR “reirradiations”[All Fields]) AND (“liver”[MeSH Terms] OR “liver”[All Fields] OR “livers”[All Fields] OR “liver s”[All Fields])) OR ((“re irradiation”[MeSH Terms] OR “re irradiation”[All Fields] OR “reirradiation”[All Fields] OR “reirradiate”[All Fields] OR “reirradiated”[All Fields] OR “reirradiating”[All Fields] OR “reirradiations”[All Fields]) AND (“carcinoma, hepatocellular”[MeSH Terms] OR (“carcinoma”[All Fields] AND “hepatocellular”[All Fields]) OR “hepatocellular carcinoma”[All Fields] OR (“hepatocellular”[All Fields] AND “carcinoma”[All Fields]))) OR ((“re irradiation”[MeSH Terms] OR “re irradiation”[All Fields] OR “reirradiation”[All Fields] OR “reirradiate”[All Fields] OR “reirradiated”[All Fields] OR “reirradiating”[All Fields] OR “reirradiations”[All Fields]) AND (“abdomen”[MeSH Terms] OR “abdomen”[All Fields] OR “abdomens”[All Fields] OR “abdominal cavity”[MeSH Terms] OR (“abdominal”[All Fields] AND “cavity”[All Fields]) OR “abdominal cavity”[All Fields])) OR ((“re irradiation”[MeSH Terms] OR “re irradiation”[All Fields] OR “reirradiation”[All Fields] OR “reirradiate”[All Fields] OR “reirradiated”[All Fields] OR “reirradiating”[All Fields] OR “reirradiations”[All Fields]) AND (“pancrea”[All Fields] OR “pancreas”[MeSH Terms] OR “pancreas”[All Fields])) OR ((“re irradiation”[MeSH Terms] OR “re irradiation”[All Fields] OR “reirradiation”[All Fields] OR “reirradiate”[All Fields] OR “reirradiated”[All Fields] OR “reirradiating”[All Fields] OR “reirradiations”[All Fields]) AND (“gastrointestinal neoplasms”[MeSH Terms] OR (“gastrointestinal”[All Fields] AND “neoplasms”[All Fields]) OR “gastrointestinal neoplasms”[All Fields] OR (“gastrointestinal”[All Fields] AND “cancers”[All Fields]) OR “gastrointestinal cancers”[All Fields]))	267
5	#1 AND #2 AND #3 AND #4	107
4	((overall[tiab] AND survival[tiab]) OR OS[tiab] OR (progression-free[tiab] AND survival[tiab]) OR PFS[tiab] OR (disease-free[tiab] AND survival[tiab]) OR DFS[tiab] OR (local[tiab] AND control[tiab]) OR (local[tiab] AND recurrence*[tiab]) OR (acute[tiab] AND toxicit*[tiab]) OR (late[tiab] AND toxicit*[tiab]) OR (adverse[tiab] AND (event*[tiab] OR effect*[tiab])) OR toxicit*[tiab] OR complicat*[tiab] OR side-effect*[tiab] OR “Treatment Outcome”[MeSH])	4,017,167
3	(“Radiotherapy”[Mesh] OR radiotherapy[Subheading] OR radiotherap*[tiab] OR “stereotactic ablative radiotherapy”[tiab] OR “stereotactic body radiotherapy”[tiab] OR SBRT[tiab] OR SABR[tiab])	434,498
2	((((((reirradiat*[tiab] OR re-irradiat*[tiab] OR reRT*[tiab] OR (repeat[tiab] AND radiotherap*[tiab]) OR (reirradiation[tiab] AND hepatocellular[tiab] AND carcinoma*[tiab]) OR (reirradiation[tiab] AND abdomen[tiab]) OR (reirradiation[tiab] AND pancreas*[tiab]) OR (reirradiation[tiab] AND gastrointest*[tiab]))) OR (reirradiation abdomen)) OR (reirradiation pancreas)) OR (reirradiation lymph node)) OR (reirradiation hepatocellular carcinoma)) OR (reirradiation liver metastas*[tiab])	4,202
1	((cancer*[tiab] OR carcinoma*[tiab] OR neoplas*[tiab] OR adenocarcinoma*[tiab] OR malignan*[tiab] OR tumor*[tiab] OR tumour*[tiab]) OR Neoplasms[MeSH]) AND ((hepatocellular[tiab] AND carcinoma*[tiab]) OR hepatocellular carcinoma OR pancreas*[tiab] OR “Pancreatic Neoplasms”[Mesh] OR (liver[tiab] AND metastas*[tiab]) OR “Liver Neoplasms”[Mesh] OR (abdominal[tiab] AND metastas*[tiab]) OR (lymph node[tiab] AND metastas*[tiab]))	473,829

**Table:** 

Section and topic	Item #	Checklist item	Reported (Yes/No)
**TITLE**			
Title	1	Identify the report as a systematic review.	Yes
**BACKGROUND**			
Objectives	2	Provide an explicit statement of the main objective(s) or question(s) the review addresses.	Yes
**METHODS**			
Eligibility criteria	3	Specify the inclusion and exclusion criteria for the review.	Yes
Information sources	4	Specify the information sources (e.g. databases, registers) used to identify studies and the date when each was last searched.	Yes
Risk of bias	5	Specify the methods used to assess risk of bias in the included studies.	N/A
Synthesis of results	6	Specify the methods used to present and synthesise results.	N/A
**RESULTS**			
Included studies	7	Give the total number of included studies and participants and summarise relevant characteristics of studies.	Yes
Synthesis of results	8	Present results for main outcomes, preferably indicating the number of included studies and participants for each. If *meta*-analysis was done, report the summary estimate and confidence/credible interval. If comparing groups, indicate the direction of the effect (i.e. which group is favoured).	Yes
**DISCUSSION**			
Limitations of evidence	9	Provide a brief summary of the limitations of the evidence included in the review (e.g. study risk of bias, inconsistency and imprecision).	Yes
Interpretation	10	Provide a general interpretation of the results and important implications.	Yes
**OTHER**			
Funding	11	Specify the primary source of funding for the review.	Yes
Registration	12	Provide the register name and registration number.	Yes

**Table:** 

SECTION AND TOPIC	Item #	Checklist item	Location where item is reported
**TITLE**			
Title	1	Identify the report as a systematic review.	1
**ABSTRACT**			
Abstract	2	See the PRISMA 2020 for Abstracts checklist.	3
**INTRODUCTION**			
Rationale	3	Describe the rationale for the review in the context of existing knowledge.	6
Objectives	4	Provide an explicit statement of the objective(s) or question(s) the review addresses.	6
**METHODS**			
Eligibility criteria	5	Specify the inclusion and exclusion criteria for the review and how studies were grouped for the syntheses.	7
Information sources	6	Specify all databases, registers, websites, organisations, reference lists and other sources searched or consulted to identify studies. Specify the date when each source was last searched or consulted.	7
Search strategy	7	Present the full search strategies for all databases, registers and websites, including any filters and limits used.	32
Selection process	8	Specify the methods used to decide whether a study met the inclusion criteria of the review, including how many reviewers screened each record and each report retrieved, whether they worked independently, and if applicable, details of automation tools used in the process.	7, 19
Data collection process	9	Specify the methods used to collect data from reports, including how many reviewers collected data from each report, whether they worked independently, any processes for obtaining or confirming data from study investigators, and if applicable, details of automation tools used in the process.	N/A
Data items	10a	List and define all outcomes for which data were sought. Specify whether all results that were compatible with each outcome domain in each study were sought (e.g. for all measures, time points, analyses), and if not, the methods used to decide which results to collect.	7
	10b	List and define all other variables for which data were sought (e.g. participant and intervention characteristics, funding sources). Describe any assumptions made about any missing or unclear information.	N/A
Study risk of bias assessment	11	Specify the methods used to assess risk of bias in the included studies, including details of the tool(s) used, how many reviewers assessed each study and whether they worked independently, and if applicable, details of automation tools used in the process.	N/A
Effect measures	12	Specify for each outcome the effect measure(s) (e.g. risk ratio, mean difference) used in the synthesis or presentation of results.	N/A
Synthesis methods	13a	Describe the processes used to decide which studies were eligible for each synthesis (e.g. tabulating the study intervention characteristics and comparing against the planned groups for each synthesis (item #5)).	N/A
	13b	Describe any methods required to prepare the data for presentation or synthesis, such as handling of missing summary statistics, or data conversions.	N/A
	13c	Describe any methods used to tabulate or visually display results of individual studies and syntheses.	N/A
	13d	Describe any methods used to synthesize results and provide a rationale for the choice(s). If *meta*-analysis was performed, describe the model(s), method(s) to identify the presence and extent of statistical heterogeneity, and software package(s) used.	N/A
	13e	Describe any methods used to explore possible causes of heterogeneity among study results (e.g. subgroup analysis, *meta*-regression).	N/A
	13f	Describe any sensitivity analyses conducted to assess robustness of the synthesized results.	N/A
Reporting bias assessment	14	Describe any methods used to assess risk of bias due to missing results in a synthesis (arising from reporting biases).	N/A
Certainty assessment	15	Describe any methods used to assess certainty (or confidence) in the body of evidence for an outcome.	N/A
**RESULTS**			
Study selection	16a	Describe the results of the search and selection process, from the number of records identified in the search to the number of studies included in the review, ideally using a flow diagram.	8, 19
	16b	Cite studies that might appear to meet the inclusion criteria, but which were excluded, and explain why they were excluded.	19
Study characteristics	17	Cite each included study and present its characteristics.	20
Risk of bias in studies	18	Present assessments of risk of bias for each included study.	N/A
Results of individual studies	19	For all outcomes, present, for each study: (a) summary statistics for each group (where appropriate) and (b) an effect estimate and its precision (e.g. confidence/credible interval), ideally using structured tables or plots.	8
Results of syntheses	20a	For each synthesis, briefly summarise the characteristics and risk of bias among contributing studies.	N/A
	20b	Present results of all statistical syntheses conducted. If *meta*-analysis was done, present for each the summary estimate and its precision (e.g. confidence/credible interval) and measures of statistical heterogeneity. If comparing groups, describe the direction of the effect.	N/A
	20c	Present results of all investigations of possible causes of heterogeneity among study results.	N/A
	20d	Present results of all sensitivity analyses conducted to assess the robustness of the synthesized results.	N/A
Reporting biases	21	Present assessments of risk of bias due to missing results (arising from reporting biases) for each synthesis assessed.	N/A
Certainty of evidence	22	Present assessments of certainty (or confidence) in the body of evidence for each outcome assessed.	N/A
**DISCUSSION**			
Discussion	23a	Provide a general interpretation of the results in the context of other evidence.	11
	23b	Discuss any limitations of the evidence included in the review.	12
	23c	Discuss any limitations of the review processes used.	12
	23d	Discuss implications of the results for practice, policy, and future research.	12
**OTHER INFORMATION**			
Registration and protocol	24a	Provide registration information for the review, including register name and registration number, or state that the review was not registered.	7
	24b	Indicate where the review protocol can be accessed, or state that a protocol was not prepared.	7
	24c	Describe and explain any amendments to information provided at registration or in the protocol.	N/A
Support	25	Describe sources of financial or non-financial support for the review, and the role of the funders or sponsors in the review.	1
Competing interests	26	Declare any competing interests of review authors.	2
Availability of data, code and other materials	27	Report which of the following are publicly available and where they can be found: template data collection forms; data extracted from included studies; data used for all analyses; analytic code; any other materials used in the review.	N/A

## Appendix B . PRISMA 2020 for abstracts Checklist

*From:* Page MJ, McKenzie JE, Bossuyt PM, Boutron I, Hoffmann TC, Mulrow CD, et al. The PRISMA 2020 statement: an updated guideline for reporting systematic reviews. BMJ 2021;372:n71. 10.1136/bmj.n71. This work is licensed under CC BY 4.0. To view a copy of this license, visit https://creativecommons.org/licenses/by/4.0/.

## Appendix C . PRISMA 2020 Checklist

*From:* Page MJ, McKenzie JE, Bossuyt PM, Boutron I, Hoffmann TC, Mulrow CD, et al. The PRISMA 2020 statement: an updated guideline for reporting systematic reviews. BMJ 2021;372:n71. 10.1136/bmj.n71. This work is licensed under CC BY 4.0. To view a copy of this license, visit https://creativecommons.org/licenses/by/4.0/.
